# Profile of resistance to IVIG treatment in patients with Kawasaki disease and concomitant infection

**DOI:** 10.1371/journal.pone.0206001

**Published:** 2018-10-17

**Authors:** Audrey Dionne, Cathie-Kim Le, Steffany Poupart, Julie Autmizguine, Léamarie Meloche-Dumas, Jean Turgeon, Anne Fournier, Nagib Dahdah

**Affiliations:** 1 Department of Cardiology, CHU Ste-Justine, Montreal, Canada; 2 Department of Cardiology, Boston Children’s Hospital, Department of Pediatrics, Harvard Medical School, Boston, MA, United States of America; 3 Department of Pediatrics, Centre Hospitalier de l’Universite Laval, Quebec, Canada; 4 Department of Pharmacology, University of Montreal, Montreal, Canada; 5 Research Center, CHU Ste-Justine, Montreal, Canada; 6 Department of pediatrics, CHU Ste-Justine, Montreal, Canada; Institut National de la Santeet de la Recherche Medicale (INSERM), FRANCE

## Abstract

**Introduction:**

Kawasaki disease (KD) can be associated with concomitant viral or bacterial infections. Children with persistent or recurrent fever 36 hours after the end of intravenous immunoglobulin (IVIG) are considered to be resistant to treatment and are at increased risk for coronary complications. Although concomitant infection does not affect coronary outcome, it is unknown how it influences the response to IVIG treatment.

**Methodology:**

Retrospective cohort study between 2008 and 2016 in a tertiary pediatric university hospital, including 154 children, of which 59 (38%) had concomitant infection.

**Results:**

Children with concomitant infection were more likely to have fever 48 hours after initial IVIG treatment (36% vs 20%, p = 0.05) and to be treated with a second dose (33% vs 18%, p = 0.04). Children with infection had higher C-reactive protein at the time of diagnosis (148 vs 112 mg/L, p = 0.04), and 48 hours after IVIG administration (111 vs 59 mg/L, p = 0.003). Nevertheless, there was no statistically significant difference in the prevalence of coronary complications (Z-score > 2.5) between children with and without concomitant infection (36% vs 39%, p = 0.68).

**Conclusion:**

Children with KD and concomitant infection are more likely to have persistent fever and elevated inflammatory markers after treatment. This association increases the likelihood of receiving a second dose of IVIG but not the risk of coronary complication. Accordingly, prospective studies to distinguish true IVIG resistance from infection induced persistent fever is warranted.

## Introduction

Kawasaki disease (KD) is an acute systemic vasculitis mostly affecting children younger than 5 years old. It is the most important cause of acquired heart disease in children in developed countries [1]. Concomitant respiratory viral infections have been described in 8–42% of patients, and bacterial infections were found in 33% of patients [2–8]. The clinical presentation of patients with and without concomitant infection is similar [2,8]. In one study, bacterial co-infection alluded to a trend towards a higher rate of resistance to intravenous immunoglobulin (IVIG), without reaching statistical significance (26% vs 15%, odds ratio 1.77 (95% confidence interval 0.71–4.41)) [8]. The accepted definition of IVIG resistance is based on the persistence or recrudescence of fever 36 hours after the end of IVIG infusion [1]. However, it is unclear how concomitant infection influences the resolution of fever and response to IVIG treatment. The aim of this study was to determine the impact of concurrent infection on the prevalence of IVIG resistance and coronary outcome.

## Methodology

### Population

This retrospective study included children with a diagnosis of KD between 2008 and 2016 followed at the Sainte-Justine’s University Hospital Center (Montreal, Canada). Inclusion criteria were a diagnosis of KD maintained at discharge based on current clinical practice and recommendations [1]; and echocardiography measurements of the coronary artery (CA) at onset, 1–2 weeks and >3 months after diagnosis. The main outcome was resistance to IVIG treatment in children with, versus those without concurrent infection. Secondary outcomes included duration of fever, progress of inflammatory markers and coronary artery complications. This study was approved by the institutional Research Ethics Committee of the CHU Sainte-Justine. The institutional Research ethics Committee waived the requirement for informed consent.

### Data collection and definitions

Medical charts were reviewed for demographic characteristics, clinical course, laboratory values and infectious workup. Clinical KD criteria were reviewed and children classified as complete or incomplete KD, in the presence of fever and ≤3 clinical criteria for the latter. Delay in IVIG administration was defined as > 10 days between onset of fever and IVIG administration; and IVIG resistance as persistent or recrudescent fever (> 38.5°C) 36 hours after the end of IVIG infusion. Concurrent infection was defined as a clinical diagnosis and proven concurrent infection as patients with a positive microbiologic testing or imaging study in the presence of clinical symptoms. Testing for concomitant infection was based on clinical symptoms. Urinary tract infection was diagnosed according to current American Academy of Pediatrics guidelines [9]. Gastroenteritis was defined as gastrointestinal symptoms with positive stool cultures for virus or pathogenic bacteria. Children with respiratory symptoms and a positive respiratory virus multiplex reverse transcription polymerase chain reaction (influenza, parainfluenza, coronavirus, enterovirus, rhinovirus) were diagnosed with upper respiratory tract infection (URTI). Otitis media was diagnosed according to current American Academy of Pediatrics guidelines [10]. Children with clinical signs of exudative pharyngitis in the presence of a positive group A streptococcus culture were diagnosed with concurrent infection. Bacterial adenitis was diagnosed in the presence of clinical symptoms with signs of abscess formation and/or necrosis on ultrasound. Children with positive serology for acute infection (IgM) with Epstein-Barr virus (EBV), cytomegalovirus (CMV), measles, parvovirus and mycoplasma before IVIG administration were considered to have concurrent infection. Pneumonia was diagnosed when chest X-ray, interpreted by a pediatric radiologist, confirmed the presence of a consolidation, in the presence of respiratory symptoms.

Echocardiographic CA size was reviewed for all children at onset of the disease, with follow-up studies for a minimum of 3 months and up to one year after diagnosis of KD. CA Z-scores were calculated for the right coronary artery, left main coronary artery, left anterior descending artery and circumflex artery. CA aneurysm was defined as a localized dilatation of a portion with adjacent normal measurements, or an obvious saccular deformation of the CA. CA dilatation of non-aneurysmal segments was defined as a CA Z-score > 2.5 [1], calculated according to Dallaire and Dahdah [11]. Early CA dilatation was defined as CA dilatation at onset up to one week following KD diagnosis, and late CA dilatation when persisting at 3 months’ follow-up.

### Statistical analysis

Quantitative variables were summarized as mean ± SD and categorical variables as frequencies and percentages. The Shapiro-Wilk test was used to test for normal distribution. Comparison of clinical and laboratory data between patients with and without concurrent infection was performed using the student’s t-test for continuous variables with normal distribution or the Mann-Whitney U test for continuous variables with non-normal distribution. ANOVA for repeated measures was used to describe the variation of temperature and laboratory values over time. The Fisher or the χ^2^ tests were used for comparison of categorical variables. Logistic regression was used to examine the association between concomitant infection and CA complications, controlling for confounder variables (IVIG resistance). All analyses were performed with SPSS Statistics version 23 (IBM, Chicago, Illinois). A two-tailed p value of <0.05 was deemed significant.

## Results

The study included 154 children (101 male; 66%) aged 3.4±2.8 years. The median number of diagnostic criteria was 5 (range 2–6), with 59 (38%) children having incomplete KD criteria. IVIG were administered to 150 (97%) patients, 6.7±2.6 days after onset of fever. Delay in IVIG administration occurred in 10 (7%) patients, and IVIG resistance in 36 (23%).

### Concomitant infection

Concurrent infections were diagnosed in 59 (38%) patients, of which 20 (34%) were viral and 39 (66%) bacterial. URTI was diagnosed in 19 (32%) children, caused by respiratory syncytial virus (n = 3), parainfluenza (n = 3), rhinovirus (n = 4), influenza (n = 2), enterovirus (n = 2) and adenovirus (n = 4). Three children had multiple viruses detected. Two (7%) children were diagnosed with viral gastroenteritis. Three (5%) children had a clinical and biological profile suggestive of acute CMV infection (positive IgM and negative IgG), and 2 (3%) of acute mycoplasma infection. Otitis media was diagnosed in 7 (12%) of patients, and pneumonia in 8 (14%) children. Group A streptococcus pharyngitis was diagnosed in 13 (22%). Four (7%) patients had bacterial adenitis, complicated by retropharyngeal pharyngitis in one child. One child was diagnosed with perforated appendicitis and underwent surgery, with pathology confirming the diagnosis. One child had Escherichia Coli pyelonephritis. Concomitant infection was proven by microbiologic testing and/or imaging in 48 (81%) of patients. Characteristics of patients with viral versus bacterial infections are described in Table 1.

**Table 1:** Characteristics of patients with viral and bacterial infection.

Patients Characteristics	Viral infection (n = 20)	Bacterial infection (n = 39)	p value
**Male** n (%)	7 (41)	18 (67)	0.10
**Age** (years)	4.1±3.1	3.2±2.8	0.28
**Incomplete clinical criteria** n (%)	13 (77)	17 (63)	0.35
**Day of fever at diagnosis** (days)	6.7±1.8	6.9±3.2	0.69
**IVIG treatment** n (%)	17 (100)	26 (96)	0.42
**IVIG delay** n (%)	0 (0)	3 (12)	0.15
**IVIG resistance** n (%)	4 (24)	12 (46)	0.13
**WBC count** (x109 cells/L)	11.8±4.4	14.8±4.9	0.08
**Neutrophils** (%)	63±17	67±14	0.57
**Hemoglobin** (g/L)	112±11	103±13	0.05
**Platelets** (x109 cells/L)	313±113	410±219	0.32
**CRP** (mg/L)	105±84	177±97	0.03
**ESR** (mm/h)	47±10	52±12	0.06
**Albumin** (g/dL)	30±5	25±6	0.07

During hospitalization, antibiotics were empirically initiated in 95 (62%) children, and completed in 36 (23%). Antibiotics were initiated on average 2±5 days prior to IVIG treatment, with antibiotics initiated prior to IVIG treatment in the majority of cases (55, 67%). Only a minority (4, 9%) were started on antibiotics after non-response to initial IVIG treatment.

Age was similar between children with and without infection (3.5±2.7 versus 3.4±2.9 years, p = 0.94), with a similar proportion of male (34 (58%) versus 67 (71%) patients, p = 0.10). There was a similar proportion of children with incomplete clinical criteria among children with and without infection (20 (34%) versus 39 (41%), p = 0.38). There were 50 (58%) children with infection who received antibiotics compared to 45 (47%) without infection who received antibiotics (p<0.001). Of the former, 29 (58%) completed antibiotic treatment versus 7 (16%) of the latter (p<0.001). Most children with and without concurrent infection received IVIG (57 (97%) and 93 (98%) patients; p = 0.63). Delay in diagnosis and IVIG administration occurred in 4 (7%) children with concurrent infection versus 6 (7%) without concurrent infection, p = 0.89 (Table 2). Finally, there was no difference in delay of IVIG administration whether patients presented with complete versus incomplete clinical criteria (7% and 7%; p = 0.93). In general, basic laboratory values were similar between children with and without concurrent infections. C-reactive protein was however higher in cases with concurrent infections compared to cases without concurrent infection (148±96 versus 112±65 mg/L, p = 0.03) (Table 3). Similar results were found between patients with proven infection on microbiologic testing and/or imaging versus those without and results are presented in Table 4.

**Table 2:** Characteristics of patients with and without concomitant infection.

Patients Characteristics	Concomitant infection (n = 59)	No concomitant infection (n = 95)	p value
**Male** n (%)	34 (58)	67 (71)	0.10
**Age** (years)	3.5±2.7	3.4±2.9	0.94
**Incomplete clinical criteria** n (%)	20 (34)	39 (41)	0.38
**Day of fever at diagnosis** (days)	6.7±2.6	6.3±2.7	0.37
**IVIG treatment** n (%)	57 (97)	93 (98)	0.63
**IVIG delay** n (%)	4 (7)	6 (7)	0.89
**IVIG resistance** n (%)	19 (33)	17 (18)	0.036

**Table 3:** Laboratory values in patients with and without concomitant infection.

Patients Characteristics	Concomitant infection (n = 59)	No concomitant infection (n = 95)	p value
**WBC count** (x109 cells/L)	13.3±5.1	14.8±5.7	0.14
**Neutrophils** (%)	64±16	64±16	0.92
**Hemoglobin** (g/L)	107±13	106±14	0.87
**Platelets** (x109 cells/L)	383±246	389±207	0.88
**CRP** (mg/L)	148±96	112±65	0.03
**ESR** (mm/h)	49±12	49±13	0.87
**Albumin** (g/dL)	27±6	27±5	0.82

**Table 4:** Characteristics of patients with and without proven infection.

Patients Characteristics	Proven concomitant infection (n = 48)	No proven concomitant infection (n = 106)	p value
**Male** n (%)	26 (54)	75 (71)	0.05
**Age** (years)	3.7±2.8	3.3±2.8	0.33
**Incomplete clinical criteria** n (%)	17 (35)	42 (40)	0.62
**Day of fever at diagnosis** (days)	6.8±2.3	6.3±2.8	0.08
**IVIG treatment** n (%)	46 (96)	104 (98)	0.41
**IVIG delay** n (%)	3 (7)	7 (7)	0.96
**IVIG resistance** n (%)	16 (35)	20 (19)	0.04
**WBC count** (x109 cells/L)	12.7±5.1	14.9±5.6	0.07
**Neutrophils** (%)	64±17	64±16	0.99
**Hemoglobin** (g/L)	107±14	106±13	0.53
**Platelets** (x109 cells/L)	365±246	397±210	0.15
**CRP** (mg/L)	130±84	125±79	0.75
**ESR** (mm/h)	48±13	49±13	0.95
**Albumin** (g/dL)	27±6	27±5	0.93

### Response to IVIG treatment

**Fig 1:**
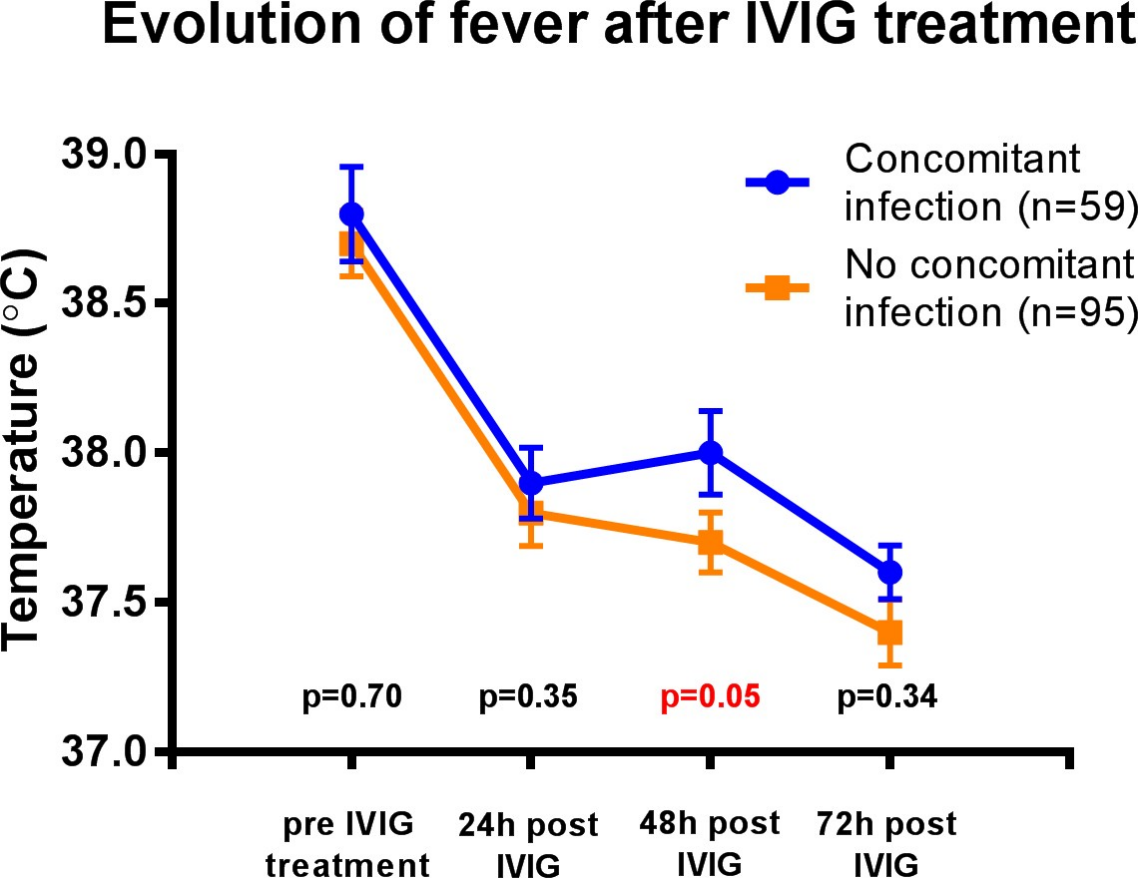
Evolution of fever after IVIG treatment in patients with and without concomitant infection. Chronological comparison of the temperature between patients with and without concomitant infection using the Mann-Whitney test. Mean ± standard error displayed on the figure.

**Fig 2:**
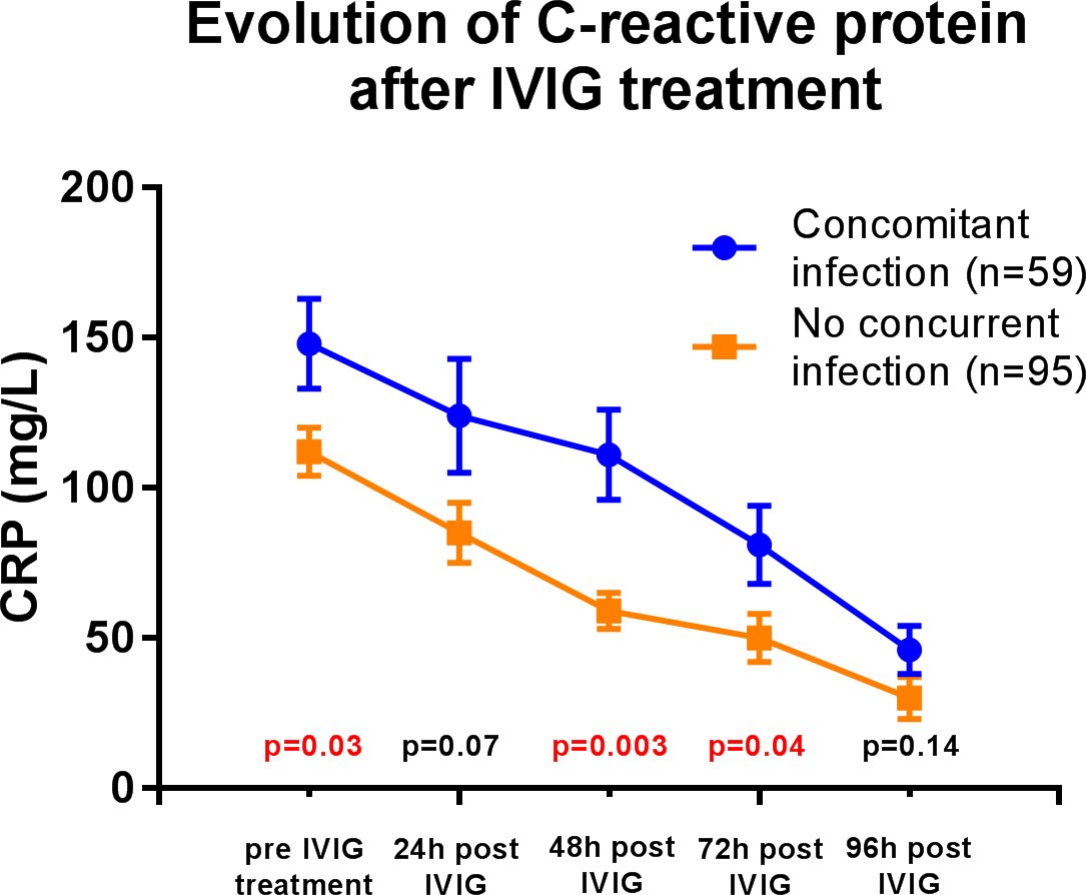
Evolution of C-reactive protein (CRP) after IVIG treatment in patients with and without concomitant infection. Chronological comparison of the CRP between patients with and without concomitant infection using the Mann-Whitney test. Mean ± standard error displayed on the figure.

Overall, IVIG resistance occurred in 36 (24%) patients. Children with concurrent infection had higher rates of IVIG resistance (19 (33%) versus 17 (18%) patients, p = 0.04), and higher temperature at 48 hours (Fig 1). They were also more likely to have fever > 38.5°C at 48 hours, than those without concurrent infection (16 (36%) versus 15 (20%) patients, p = 0.05). This was accompanied with higher CRP at time of diagnosis, remaining similarly higher in the first 72 hours after treatment (Fig 2).

IVIG resistance was higher in patients with proven infection on microbiologic testing and/or imaging than those without (16 (35%) versus 20 (19%) patients, p = 0.04). There was no difference in response to treatment between patients who had a proven infection on microbiologic testing and/or imaging study versus those with clinical diagnosis of infection (16 (35%) versus 3 (27%) patients, p = 0.64). Resistance to initial IVIG treatment in patients with bacterial infection was nearly double that in patients with viral infection, although not reaching statistical significance (12 (46%) versus 4 (24%) patients, p = 0.12). There was no difference in response to treatment between patients with complete and incomplete clinical criteria (25 (28%) versus 10 (17%) patients, p = 0.12).

Patients who received antibiotics during their hospital course, independent of infection status, were at higher risk of IVIG resistance than those who did not (27 (29%) versus 9 (16%) patients, p = 0.05). However, neither receiving antibiotics prior to IVIG therapy (15 (27%) versus 11 (41%) patients, p = 0.22) nor completing antibiotic course (13 (36%) versus 14 (25%) patients, p = 0.25) were associated with response to treatment.

## Coronary outcome

**Fig 3:**
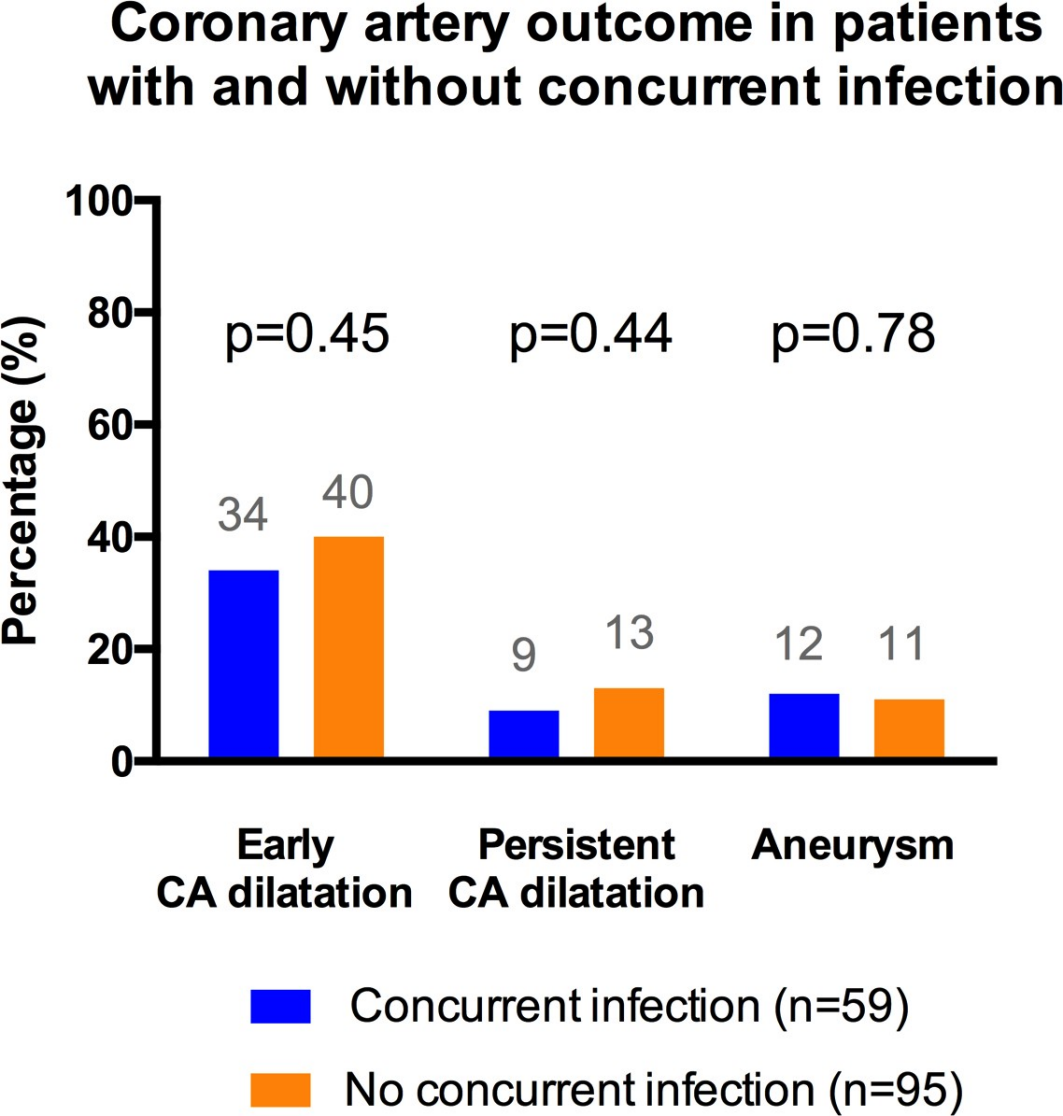
Coronary artery outcome in patients with and without concomitant infection. Comparison between patients with and without concomitant infection for each category of coronary involvement using the chi square test.

There was no significant difference in coronary artery complications (Fig 3) between patients with and without infection (21 (36%) versus 37 (39%), p = 0.68). This remained true even after adjusting for IVIG resistance and number of IVIG treatment (p = 0.47). Resistance to IVIG treatment was associated with an increased risk of CA complication both as a univariate (53% versus 34%, p = 0.037), and when adjusting for the presence of infection (p = 0.039). Coronary artery dilation at time of diagnosis was found in 58 (38%) patients, similarly distributed according to the presence or absence of concurrent infection (20 (34%) versus 38 (40%) patients, respectively; p = 0.45), and persisted in 17 (11%) patients (in 5 (9%) versus 12 (13%) patients, respectively; p = 0.42). Coronary aneurysms were diagnosed in 17 (11%) patients, without significant difference between patients with and without infection neither (7 (12%) versus 10 (11%) patients, p = 0.80). While the risk of coronary aneurysm was similar between patients with viral versus bacterial infection (5 (19%) versus 2 (12%) patients, p = 0.69), patients with bacterial infection were more likely to have coronary artery dilation (13 (48%) versus 3 (18%) patients, p = 0.04).

## Discussion

In this retrospective series, the presence of a concomitant infection was associated with a higher rate of resistance to IVIG treatment. Patients with concomitant infection were more likely to have persistent fever and slower normalization of inflammatory markers after IVIG treatment. The administration of antibiotics did not decrease this risk of resistance to IVIG therapy. However, concomitant infection was not associated with an increased risk of coronary artery complications.

More precisely, the higher likelihood of repeated IVIG treatment when concurrent infection was present is defined as recrudescence of fever 36 hours after the end of IVIG infusion based on expert opinion [1]. Our results challenge this definition since fever may be maintained by an infection, either viral or bacterial, that is not likely to respond to IVIG. Inflammatory markers are often used to help guide the decision about the need for additional treatment in KD. In our series, patients with concurrent infection presented higher CRP levels at baseline, as well as 48 hours after initial treatment. This is consistent with a retrospective study on the variability in response to IVIG, which showed a higher rate of concomitant infection and higher CRP levels in complete non-responders to IVIG treatment compared to partial non-responders and responders [12]. Thus, both the persistence of fever after IVIG treatment and elevated inflammatory markers contribute to the increased likelihood of “IVIG resistance” in patients with concomitant infections. Interestingly, non-response to IVIG therapy was associated with the use of antibiotics in a prior single-center retrospective study [12]. One would have expected patients with concomitant infection, at least bacterial, to have lower rate of IVIG resistance if the infection was controlled by antibiotics. Both in prior reports and this series [12], the use of antibiotic was found to be associated with non-response to IVIG treatment, independent of whether or not an infection was confirmed. However, it is not clear if it is just reflecting the underlying infection, or KD that is resistant to initial treatment.

Another hypothesis would be that the presence of infection triggers an additional inflammatory response at the molecular levels that impact response to treatment and outcomes. IL-1α and IL-1β have been shown to be essential in the development of KD [13]. The IL-1 pathways play a critical part of the host defense against microbial pathogens through activation of toll like receptors [14,15]. Moreover, inositol-triphosphate 3-kinase C (ITPKC) has a critical role in mediating NLRP3 expression and intracellular calcium, which is then responsible for IL-1β production [16]. Genetic polymorphism in ITPKC is associated with higher IL-1β cytokines and treatment failure [16]. This raises the question if concomitant infection increases resistance to IVIG treatment by increasing levels of IL-1β cytokines. This could be very important as new treatment strategies are targeting IL-1 blockade in recalcitrant KD.

Persistence of fever after IVIG treatment is a strong risk factor for development of coronary aneurysms [1,17]. In this series, patients with resistance to IVIG treatment had a higher risk of developing CA complication. Notwithstanding, the rate of CA complication was statistically independent of the presence of infection in our series and in previous series [8]. Thus, the lack of increased risk of CA complication in the setting of persistent fever in patients with KD and concomitant infection argues for infection as the cause of fever as opposed to IVIG resistance. It questions whether this “excessive” diagnosis of IVIG resistance is well justified when there is a concomitant infection, and if the current definition of treatment resistance should be modified. Retreatment with IVIG or other immunosuppressive therapies may also have had a protective effect on the development of CA complication. However, the rate of CA complication was similar between patients with and without infection, even after accounting for the number of IVIG treatment received. Thus, other criteria need to be used to help with decision for retreatment of patients with persistent fever and concomitant infection. Coronary artery dimensions may be a more useful marker for the need for additional treatment in this at risk population.

In general, patients who are resistant to initial treatment receive a second dose of IVIG (2 g/kg) [18]. Notwithstanding, at least 5% of children remain febrile despite multiple dose of IVIG [18]. These particular patients are at even greater risk of CA complications, and additional therapies are usually administered [19], including corticosteroids [20–22], anti-TNF alpha agents [23,24], cyclosporine [25], cyclophosphamide [26] and more recently anakinra (anti-interleukine1) [27,28]. The use of these additional therapies is based on effectiveness in other vasculitis, with no prospective clinical trials to show effectiveness on coronary artery outcome. In patients with concerns for concurrent infection, the benefit of those additional therapies should be carefully balanced against the increased risk of infection. It is important to make this distinction, in order to 1) avoid using aggressive immunosuppressive therapies in patients with persistent fever due to uncontrolled infection and 2) avoid not treating aggressively a patient with KD that is resistant to initial treatment and at increased risk of serious coronary artery complications. However, this distinction can be clinically very difficult to answer, and caution should be exercise to prevent both cardiac complications and adverse side effects of therapies.

There are limitations to this study essentially related to the retrospective methods, and the diagnosis of concomitant infection. On one hand, in the absence of systematic testing for infections there is a potential underestimation of the actual concurrent infection rate, as some infections could not be identified. On the other hand, inclusion of only children with infectious workup at time of initial KD diagnosis could have falsely increased the rate of concurrent infection. However, the rate of concomitant infection in this series was similar to those previously published [8]. Moreover, positive testing for virus and/or throat culture in children could reflect a carrier status rather than actual concurrent infection. However, because infectious workup was performed based on children’s symptoms, the positive results most likely represent an actual infection. Serologies for different viral infections (CMV, EBV, mycoplasma) can be difficult to interpret in an acute inflammatory setting, with IgM cross-reactivity. These infections could not be confirmed by PCR due to the retrospective nature. However, this could only have affected 3 children, and statistical analysis excluding these children did not affect the results. Moreover, whereas the AHA definition of resistance to IVIG treatment is based on persistent fever 36 hours after the end of IVIG infusion [1], other factors are considered in the decision whether or not to retreat patients, including inflammatory markers and CA dimensions. Thus, some patients were classified as resistant to treatment based on the definition, but were not retreated and had favorable evolution. Small sample size of sub-groups analysis and secondary aims limit the statistical power, and results should be interpreted in light of these limitations.

## Conclusion

In this study, patients with concomitant infection had a higher rate of resistance to IVIG treatment. Patients with concomitant infection had longer duration of fever and slower normalization of inflammatory markers after initial treatment. However, the presence of infection was not associated with an increased risk of CA complication. Accordingly, the persistence of fever in KD and the definition of resistance to IVIG should be regarded speculatively when concurrent infection is present. Decision to intensify treatment in patients with concurrent infection should not only be based on persistent fever. Coronary artery dimensions may be a more useful indication for treatment in this patient population. Prospective studies are needed to better refine which children truly require additional therapies.

## Abbreviations

CA: Coronary artery
CMV: Cytomegalovirus
EBV: Epstein-Barr virus
IVIG: Intravenous immunoglobulins
KD: Kawasaki disease
URTI: Upper respiratory tract infection
